# Consumption—Its New Cure

**Published:** 1859-02

**Authors:** 


### HALL'S JOURNAL OF HEALTH.

OUR LEGITIMATE SCOPE IS ALMOST BOUNDLESS: FOR WHATEVER BEGETS PLEASURABLE AND HARMLESS FEELINGS, PROMOTES HEALTH; AND WHATEVER INDUCES DISAGREEABLE SENSATIONS, ENGENDERS DISEASE.

*We aim to show how Disease may be avoided, and that it is best, when sickness comes, to take no Medicine without consulting an educated Physician.*

VOL. VI.] FEBRUARY, 1859. [No. 2.

### CONSUMPTION—ITS NEW CURE.

Men of intelligence and reflection are falling into the habit of requiring something more of the physician than his advice and his medicine. They have a curiosity to know what the remedy is, and how it is expected to effect a cure. Within the last few months millions of people have been made acquainted with a very hard word, with the previous existence of which they perhaps never had any knowledge. But it is often desirable that men of an inquiring turn of mind should extend the circle of their acquaintance, &c. "Hypophosphite" has been introduced into very many families, and received with a welcome; the other part of the name is lime. It reads in full thus: "Hypophosphite Lime," and is claimed to have ability to treat successfully scrofula, consumption of the bowels, and consumption itself. The words run thus: "The cure of consumption in the second and third (the last, Ed.) stages, except when the existing lesion of the lungs is of itself sufficient to produce death." That is, "cures consumption in all cases where there are lungs enough left to live upon." It was reported, at the time of General Jackson's death, that on the examination of the body it was found that one-third of his lungs had been destroyed, and that there was conclusive evidence that such destruction had been occasioned twenty years before. If this be true, then it follows that a man who has two-thirds of his lungs left may live twenty years in reasonable health. Therefore, "Hypophosphite Lime" can cure

" all cases" of consumption if only one-third of the lungs are destroyed.

Now, as the lungs of a good-sized man hold (that is, measure) two hundred and fifty cubic inches of air—or, in other words, can emit, after one full breath, about six tincupfulls of air, if in good health, it follows that if he has consumptive symptoms, be they ever so aggravated, if he is still able to measure, to expire four pints or two quarts or half a gallon, he can " in all cases" be cured by HYPOPHOSPHITE LIME, M. D., ESQ. Any person, then, who is in the latter stages of consumption, must take two steps preparatory to discovering one more essential; one is merely for " satisfaction," and the other indispensible, first pay us a fair fee, according to his ability, for finding to the fraction of an inch, before his own eyes, and to his full satisfaction, how much air his lungs measure out, which we can do in two minutes, with mathematical demonstrability, and then if he can, at one full outbreathing, emit one hundred and sixty-six and two-third inches of air, and Hypophosphite Lime will cure " in all cases."

How do we know that? " Why, all the papers say so;" and that is conclusive enough of its truth in the estimation of a good many people. This being fixed, how will the cure be effected? We will now drop all round abouts, premising that oil of vitriol be poured on some burnt bones, and the ashes of seaweed be stirred in (oil of vitriol is powerful, and anything that has " sea" attached to it has great health properties in the estimation of every body,) and then allowed to settle, pour off, then pour on boiling water, stir, let settle, pour off, and dry the remnant, and we will have in the shape of the purest whitest powder a pretty good idea of the Hypophosphite of Lime and Soda. As much of this as will rest on a twenty-five cent piece, taken daily in sweetened water, one-third at a time, is the curer of consumption in its last stages, if two-thirds of the lungs are left. How?

We know that the human body has bones in it. We know that healthy bones contain phosphorus. We know that in consumption the bones have not enough of phosphorus.

All this is plain sailing. The next step, however, brings us right jam up against a mountain of brass; you can't look it out of countenance, for the looker gets out of countenance instead of the lookee, from being reminded of the fact how little he knows. For example, we do not know what other things besides phosphorus the system needs when in a consumptive condition. The most learned chemists and physiologists have not been able to decide whether phosphorus exists in the system with oxygen in it, or with none—that is, we don't know in what shape the system needs phosphorus, nor whether it is to be had outside the body in the shape in which the body will take hold of it and appropriate it to building purposes. Dr. Gregory, of Edinburgh, says it is absurd to suppose that it can exist in the body without oxygen; but Dr. Churchill, on the ground that Dr. Gregory is entirely wrong, "deduced" that if given to the body in the shape in which it combines oxygen with itself, it would cure consumption; and, as the Hypophosphite of Lime fulfils that condition, he advocates its employment.

Thus it is that the very theory that Hypophosphites are good in consumption is founded on assuming as a fact what eminent men strongly deny.

But, without wasting time in discussing mere theories, practical men have put the matter to a direct test, and have reported that the Hypophosphites of Lime and Soda are of no curative value whatever in consumption; that the least that can be said of them is—they neither do good nor harm—but, if anything, they do harm by the loss of time in using them, which might have been better employed in other ways. We therefore repeat the assertion of our last number, that the best things to take in any and all cases of consumption are exercise, substantial food, and out-door air in large but due proportions, and that without these no case of consumptive disease has ever been successfully treated by any man, living or dead.

